# Maternal venous hemodynamics in gestational hypertension and preeclampsia

**DOI:** 10.1186/1471-2393-14-212

**Published:** 2014-06-23

**Authors:** Wilfried Gyselaers, Kathleen Tomsin, Anneleen Staelens, Tinne Mesens, Jolien Oben, Geert Molenberghs

**Affiliations:** 1 Department of Obstetrics & Gynaecology, Ziekenhuis Oost, Limburg Schiepse Bos 6, 3600 Genk, Belgium; 2 Department of Physiology, Hasselt University, Diepenbeek, Belgium; 3 Department of Medicine and Life Sciences, Hasselt University, Diepenbeek, Belgium; 4 Center for Medical Statistics, Hasselt University, Diepenbeek, Belgium

**Keywords:** Hemodynamics, Pregnancy, Hypertension, Preeclampsia

## Abstract

**Background:**

To evaluate characteristics of venous hemodynamics, together with cardiac and arterial function, in uncomplicated pregnancies (UP), non-proteinuric gestational hypertension (GH) and preeclampsia (PE).

**Methods:**

In this observational cross-sectional study, venous hemodynamics was assessed using a standardised protocol for combined electrocardiogram (ECG)-Doppler ultrasonography, together with a non-invasive standardised cardiovascular assessment using impedance cardiography (ICG) in 13 women with UP, 21 with GH, 34 with late onset PE ≥ 34 w (LPE) and 22 with early onset PE < 34 w (EPE). ECG-Doppler parameters were impedance index at the level of hepatic veins (HVI) and renal interlobar veins (RIVI) together with venous pulse transit times (VPTT), as well as resistive and pulsatility index, and arterial pulse transit time (APTT) at the level of uterine arcuate arteries. ICG parameters were aortic flow velocity index (VI), acceleration index (ACI) and thoracic fluid content. Mann Whitney U-test, Kruskall-Wallis test and linear regression analysis with heteroskedastic variance was used for statistical analysis.

**Results:**

RIVI in both kidneys was >15% higher (P ≤ .010) in LPE and EPE, as compared to GH and UP. Next to this, >30% lower values for VI and ACI (P ≤ .029), and > 15% lower values for APTT (P ≤ .012) were found in GH, LPE and EPE, as compared to GH.

**Conclusion:**

In comparison to UP, similar abnormalities of central arterial function and APTT were found in GH, EPE and LPE. Proteinuria of LPE and EPE was associated with increased RIVI, this was not observed in GH.

## Background

Abnormal arterial tone and increased arterial stiffness are well known features of preeclampsia [1,2]. This is reflected in abnormal notching and increased pulsatility index in duplex ultrasound assessment of the uterine arteries [3] and in abnormal serum analytes in a subpopulation of pregnant women destined to develop preeclampsia [4]. It has been reported that the maternal heart is subject to morphological and functional changes during uncomplicated pregnancy [5] and that this cardiac adaptation is different in women with preeclampsia [6,7] or destined to develop preeclampsia [8]. Next to this, it has also been reported that preeclampsia is associated with venous hemodynamic dysfunction [9], which is present weeks before clinical onset of disease in early onset (EPE) but not late onset (LPE) preeclampsia [10,11].

Cardiovascular profiling is defined as the integrated assessment of an individual’s cardiac and vascular function, which was reported to differ between EPE and LPE [12]. It has been suggested that the addition of venous hemodynamic function assessment to the maternal cardiovascular profile may be useful in the assessment of women with gestational hypertensive disease [10].

Combined electrocardiogram - Doppler ultrasonography (ECG-D) [13-15] and impedance cardiography (ICG) are non-invasive methods to study maternal hemodynamics, with acceptable reproducibility and repeatability when applied according to standardized protocols [16-20].

In this study, we used ECG-D evaluation of maternal arterial and venous hemodynamics, and ICG for assessment of cardiac and arterial function, to evaluate the cardiovascular profile of women with uncomplicated pregnancy (UP), non-proteinuric gestational hypertension (GH), early- (EPE) or late-onset preeclampsia (LPE).

## Methods

Approval of the local Committee for Medical Ethics was obtained before study onset (MEC ZOL reference 08/049, 09/049 and 09/050). The study was performed conform the declaration of Helsinki.

Women, admitted between 1/10/2009 and 31/10/2012 to the Fetal Maternal Medicine Unit of Ziekenhuis Oost Limburg Genk Belgium for gestational hypertensive disease, were included. For this study, we excluded women with multiple gestation, essential hypertension, renal disease, history of organ transplantation, women with concomitant diseases as diabetes, thyroid dysfunction, cholestasis, or liver disease, and women with signs of atypical preeclampsia, such as HELLP syndrome without proteinuria or with non-hypertensive proteinuria, leaving for inclusion only those women without known diseases and new onset hypertension > 20 weeks. Definitions of gestational hypertension and preeclampsia were used, according to the criteria of the National High Blood Pressure Education Program Working Group on High Blood Pressure in Pregnancy [21]: hypertension is defined as blood pressure > 140/90 mm Hg on at least 2 occasions at least 6h apart. New onset hypertension > 20 weeks with proteinuria < 300 mg/24h is defined as gestational hypertension (GH), whereas hypertension with proteinuria ≥ 300 mg/24h is defined as preeclampsia (PE). PE < 34 weeks is defined as early onset preeclampsia (EPE), whereas PE ≥ 34 weeks is defined as late onset preeclampsia (LPE).

All women had clinical observations for at least 24h, including blood pressure measurement 3 times a day, fetal monitoring 1pd, 24h urine sampling for quantitative assessment of creatinine clearance and proteinuria, and serum analysis for hemoglobin (Hb) level, hematocrit (Hct), blood platelet count and concentrations of AST, ALT and uric acid. After informed consent, all women had ECG-D investigations in supine position at the level of hepatic veins (HV), renal interlobar veins (RIV) of both kidneys, and left and right arcuate uterine arteries as reported [22], using a 3.5 – 5 MHz probe (Toshiba Aplio Mx^©^, Toshiba Medical Systems nv, Sint-Stevens-Woluwe, Belgium). Uterine arcuate arteries were preferred over uterine arteries for their intra-parenchymatous localization, comparable with the localization of hepatic and renal interlobar veins [15]. The Doppler ultrasound measurements of the uterine arcuate arteries were performed within a maximum of 2 cm distance from the bifurcation at the uterine artery. None of the subjects were in labor at the time of the investigations. Doppler investigations were performed by sonographers, with known inter-observer and intra-observer correlations of ≥ 0.80. Care was taken to perform as much as possible all Doppler examinations before the initiation of antihypertensive therapy.

Maternal Doppler flow parameters were classified as venous impedance index (RIVI and HVI for RIV and HV respectively) or arterial resistivity index (RI), defined as (maximum velocity – minimum velocity)/maximum velocity. Arterial pulsatility index (PI) was defined as (maximum velocity – mean velocity)/maximum velocity. Maternal venous pulse transit times (VPTT) were defined as the time interval (ms) between the maternal ECG P-wave and corresponding Doppler A-wave, corrected for the duration of the corresponding cardiac cycle [14]. Maternal arterial pulse transit time (APTT) was defined as the time interval (ms) between the maternal ECG Q-wave and start of Doppler systole, again corrected for the duration of the corresponding cardiac cycle [15].

Immediately after the Doppler ultrasonography, all women had Impedance Cardiography (ICG) assessment in supine and standing position using the Non-Invasive Continuous Cardiac Output Monitor (NICCOMO^©^, Software version 2.0, Medis Medizinische Messtechnik GmbH, Ilmenau Germany) according to the reported methodology and protocol with known repeatability [17]. This impedance technique is a non-invasive method of evaluating haemodynamic parameters, based on thoracic resistance changes measured during each heart cycle of a high frequent, low powered electrical current using a set of 4 skin-electrodes [16,23].

ICG-parameters were classified as pressure parameters, left ventricular output parameters, aortic flow parameters, and thoracic fluid content (TFC in 1/kOhm) [16,17]. Pressure parameters were systolic blood pressure (SBP), diastolic blood pressure (DBP), pulse pressure (PP = SBP – DBP) and mean blood pressure (MBP = DBP + PP/3), all expressed in mmHg. Left ventricular output parameters were stroke volume (SV) in mL, heart rate (HR) in beats/min and cardiac output in mL/min (CO = HR × SV). Aortic flow parameters were velocity index (VI in 1/1000/s) which is equivalent to the amplitude of the systolic wave, acceleration index (ACI in 1/100/s^2^) which stands for the maximum acceleration of blood flow in the aorta, Heather index (HI in Ohm/s^2^) in which the amplitude of the systolic ICG wave is corrected for the time needed by the ventricle to reach maximum ejection, and total arterial compliance (TAC in mL/mmHg) which is calculated as SV/PP and is equivalent for distensibility of the arterial vascular system [16].

ICG and maternal Doppler flow parameters were also collected at 38 weeks of gestation in a total of 13 women with confirmed normal outcome of pregnancy.

All women delivered in Ziekenhuis Oost-Limburg Genk Belgium, and data on maternal and neonatal outcome were collected after delivery: maternal age and body mass index at admission (weight in kg/length in m^2^), parity, gestational age at observation and delivery (weeks), birth weight (g) and birth weight percentiles according to population specific reference values [24].

Data were filled into a database and categorized as (1) UP, (2) GH, (3) LPE and (4) EPE, and expressed as means and interquartile ranges. SPSS software version 20.0 was used for statistical comparison at nominal level α = 0.05, using Mann–Whitney U-test for comparison between individual groups and Kruskall-Wallis test for combined comparison of groups. Significant linear dependence between clinical, laboratory, ICG and ECG-Doppler variables was identified using Pearson’s correlation coefficient (PCC) at nominal level α = 0.05 (two-tailed), and goodness of fit of the resulting linear regression model was reported by R^2^ and corresponding p-value. Correlations were calculated between VPTT and venous impedance index for UP, GH, LPE and EPE at the level of HV and RIV, and compared statistically using linear regression analysis with heteroskedastic variance (SAS software V9.2).

## Results

A total of 90 women were included: 13 with UP, 21 with GH, 34 with LPE and 22 with EPE.

Table 1 lists the demographic characteristics in the 4 patient groups. Maternal age, BMI, number of smokers and nulliparity were not different between groups. The same was true for values of Hb, Hct, platelet count, AST, ALT, and creatinine clearance. Gestational age at delivery, birth weight and birth weight percentile were different between groups, with lowest values for EPE. Concentrations of serum uric acid and proteinuria were highest for EPE and lowest for GH. Antihypertensive medication use in all groups was less than 10%.

**Table 1:** Maternal characteristics, pregnancy outcome and laboratory results

		**UP (n = 13)**	**GH (n = 21)**	**LPE (n = 34)**	**EPE (n = 22)**	***P*-value (Kruskall-Wallis) (UP to EPE)**
*Maternal characteristics*	*Age (years)*	31 (28; 32)	32 (30; 36)	29 (26; 34)	29 (26; 30)	.058
	*BMI*	23 (21; 26)	24 (22; 29)	27 (23; 34)	25 (24; 31)	.103
	*Nulliparity (n, %)*	6 (46)	15 (71)	28 (82)	17 (77)	.088
	*Smokers (n, %)*	0 (0)	2 (10)	3 (9)	3 (14)	.524
*Pregnancy outcome*	*Gest. Age at delivery (weeks)*	40 (38; 41)	39 (39; 40)	38 (37; 39)	32 (29; 34)	<.001
	*Birth weight (g)*	3455 (3180; 3790)	3545 (3120; 3743)	3030 (2749; 3679)	1607 (1176; 1933)	<.001
	*Birth weight (percentile)*	50 (38; 79)	63 (31; 83)	38 (18; 75)	31 (18; 50)	.007
*Lab results*	*Hb (g/dL)*	/	12.1 (11.4; 13.3)	11.7 (10.6; 12.5)	12.1 (11.2; 13.2)	.248
	*Hct (%)*	/	35.4 (33.6; 38.4)	34.2 (31.9; 36.4)	35.6 (33.1; 36.9)	.317
	*Blood platelets (1000/μL)*	/	193 (152; 227)	183 (165; 256)	178 (142; 218)	.632
	*AST (U/L)*	/	19 (16; 22)	18 (15; 25)	19 (16; 32)	.326
	*ALT (U/L)*	/	12 (10; 14)	13 (10; 20)	15 (10; 34)	.205
	*UrAc (mmol/L)*	/	0.129 (0.106; 0.163)	0.155 (0.129; 0.178)	0.171 (0.137; 0.199)	.014
	*Prot24h (mg)*	/	174 (134; 222)	858 (388; 2273)	2254 (899; 5345)	<.001
	*CreatCl (mL/min)*	/	124 (92; 149)	125 (97; 155)	119 (91; 152)	.898

Maternal characteristics, pregnancy outcome and laboratory results in women with uncomplicated pregnancy (UP), gestational hypertension (GH), late- and early-onset preeclampsia (LPE and EPE, respectively). BMI = body mass index, Hb = hemoglobin concentration, Hct = hematocrit, AST = aspartate transaminase, ALT = alanin transaminase, UrAc = serum Uric Acid concentration, Prot24h = 24 h proteinuria, CreatCl = creatinine clearance.

Table 2 shows the maternal ICG measurements in the 4 groups, which were measured at 38 (37;40) weeks in UP, GH and LPE and at 32 (29;34) weeks in EPE. In the gestational hypertension groups, blood pressures were higher than in UP as expected, but also aortic flow parameters ACI, VI and TAC were lower than in UP. Thoracic fluid content was lower in GH than in other groups.

**Table 2:** Blood pressures and ICG parameters

		**UP (n = 13)**	**GH (n = 21)**	**LPE (n = 34)**	**EPE (n = 22)**	***P*-value (Kruskall-Wallis) (UP to EPE)**
*Gestational age*		38 (38; 39)	38 (37; 39)	38 (36; 39)	32 (30; 34)	<.001
*Pressures*	*SBP (mmHg)*	124 (117; 132)	150 (138; 161)	155 (141; 168)	155 (145; 172)	<.001
	*DBP (mmHg)*	84 (81; 88)	98 (92; 104)	102 (96; 109)	101 (92; 109)	<.001
	*MAP (mmHg)*	95 (92; 99)	111 (102; 117)	115 (108; 120)	113 (107; 123)	<.001
	*PP (mmHg)*	38 (36; 44)	53 (44; 57)	53 (41; 64)	57 (49; 63)	<.001
*Left ventricular output*	*CO (L/min)*	7.6 (6.5; 8.9)	7.9 (7.0; 9.4)	8.4 (7.1; 10.3)	7.7 (6.6; 9.1)	.359
	*HR (1/min)*	95 (81; 100)	94 (92; 102)	90 (82; 101)	96 (79; 105)	.634
	*SV (mL)*	84 (77; 96)	89 (69; 98)	91 (79;114)	85 (68; 95)	.352
*Aortic flow*	*VI (1/1,000/s)*	67 (50; 69)	44 (41; 61)	44 (36; 54)	46 (34; 61)	.018
	*ACI (1/100/s*^*2*^*)*	117 (76; 142)	75 (60; 100)	73 (56; 87)	77 (56; 111)	.043
	*HI (Ohm/s*^*2*^*)*	15.5 (10.6; 20.7)	12.3 (10.4; 16.6)	11.1 (7.6; 13.9)	10.9 (6.4; 15.4)	.066
	*TAC (mL/mmHg)*	2.1 (1.9; 2.6)	1.6 (1.3; 2.3)	1.7 (1.4; 2.4)	1.5 (1.2; 1.8)	.019
*Thoracic fluid content*	*TFC (1/kOhm)*	31.9 (27.7; 34.9)	27.5 (26.6; 30.2)	32.3 (29.2; 36.0)	34.9 (32.1; 40.6)	<0.001

Parameters of maternal cardiovascular function as measured by impedance cardiography (ICG) in uncomplicated pregnancy (UP), gestational hypertension (GH), late- and early-onset preeclampsia (LPE and EPE, respectively). SBP = systolic blood pressure, DBP = diastolic blood pressure, MAP = mean arterial pressure, PP = pulse pressure, CO = cardiac output, HR = heart rate, SV = stroke volume, VI = aortic flow velocity index, ACI = aortic flow acceleration index, TAC = total arterial compliance, TFC = thoracic fluid content.

As shown in Table 3, combined ECG-Doppler renal VPTT and uterine arcuate APTT, and RIVI, RI and PI were different between groups, with respectively lowest and highest values for EPE. Only hepatic VPTT was not different between groups.

**Table 3:** Combined ICG-Doppler parameters

			**UP (n = 13)**	**GH (n = 21)**	**LPE (n = 34)**	**EPE (n = 22)**	***P*-value (Kruskall-Wallis) (UP to EPE)**
*Gestational age*			38 (38; 39)	38 (37; 39)	38 (36; 39)	32 (28; 33)	<.001
*Arterial*	*Resistivity and pulsatility*	*Left RI*	0.54 (0.43; 0.60)	0.56 (0.39; 0.62)	0.56 (0.48; 0.66)	0.75 (0.60; 0.81)	.001
		*Left PI*	0.75 (0.55; 0.85)	0.82 (0.49; 0.90)	0.79 (0.63; 1.01)	1.19 (0.84; 1.35)	.001
		*Right RI*	0.60 (0.51; 0.68)	0.46 (0.39; 0.56)	0.55 (0.46; 0.61)	0.64 (0.51; 0.78)	.005
		*Right PI*	0.87 (0.62; 1.05)	0.58 (0.49; 0.78)	0.77 (0.61; 0.88)	0.94 (0.69; 1.26)	.006
	*Pulse transit time*	*Left APTT*	0.36 (0.31; 0.40)	0.30 (0.28; 0.34)	0.28 (0.23; 0.30)	0.26 (0.22; 0.30)	<.001
		*Right APTT*	0.39 (0.37; 0.41)	0.30 (0.29; 0.35)	0.28 (0.24; 0.30)	0.26 (0.23; 0.30)	<.001
*Venous*	*Impedance index*	*Left RIVI*	0.40 (0.35; 0.42)	0.35 (0.31; 0.43)	0.43 (0.36; 0.47)	0.45 (0.37; 0.53)	.010
		*Right RIVI*	0.34 (0.30; 0.42)	0.31 (0.27; 0.36)	0.38 (0.32; 0.43)	0.44 (0.38; 0.49)	.001
		*HVI*	0.19 (0.10; 0.60)	0.27 (0.08; 0.49)	0.27 (0.15; 0.67)	0.74 (0.23; 1.55)	.044
	*Pulse transit time*	*Left VPTT*	0.47 (0.42; 0.52)	0.43 (0.39; 0.45)	0.39 (0.36; 0.45)	0.34 (0.24; 0.45)	.003
		*Right VPTT*	0.41 (0.37; 0.50)	0.40 (0.36; 0.46)	0.38 (0.35; 0.43)	0.34 (0.29; 0.40)	.043
		*Liver VPTT*	0.39 (0.34; 0.54)	0.29 (0.21; 0.36)	0.33 (0.22; 0.44)	0.29 (0.19; 0.43)	.065

Combined electrocardiogram (ECG)-Doppler assessment of arterial hemodynamics at left and right uterine arcuate arteries and of venous hemodynamics at left kidney, right kidney and liver. Arterial resistivity index (RI) is defined as (maximum velocity – minimum velocity)/maximum velocity). RI is the equivalent to venous impedance index, which at the level of renal interlobar veins is called RIVI, and at the level of hepatic veins is called HVI. Arterial pulsatility index is defined as (maximum velocity – mean velocity)/maximum velocity), VPTT is the venous pulse transit time and APTT is the arterial pulse transit time.

**Figure 1:**
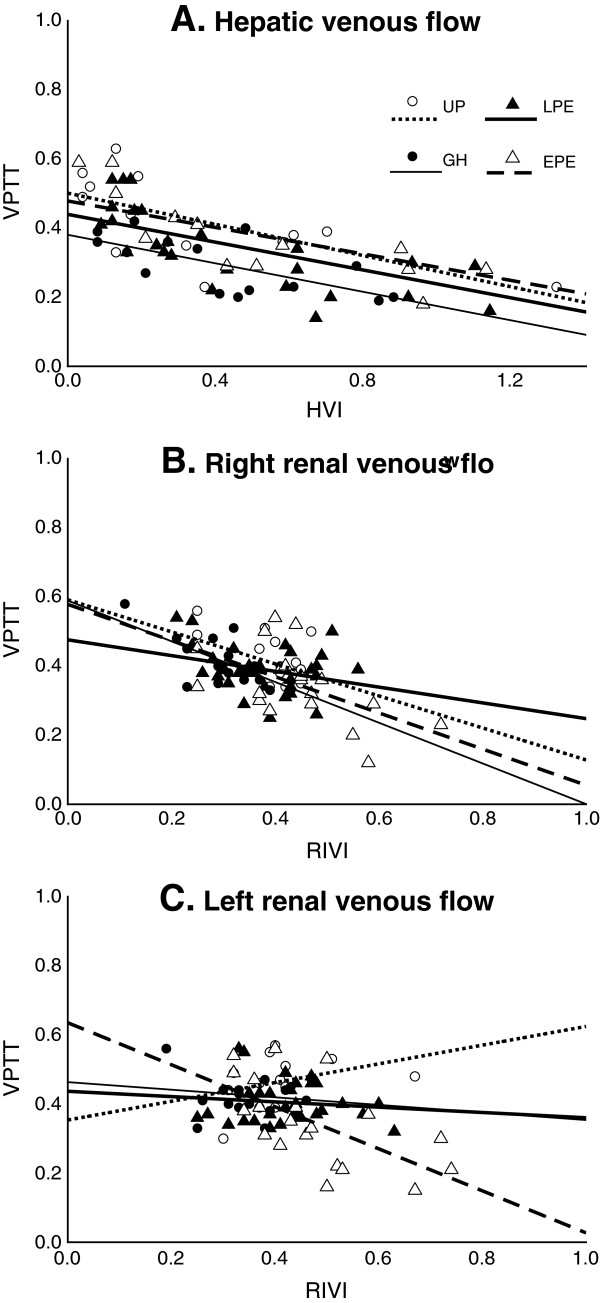
**Correlation between venous pulse transit time and venous impedance index in liver and kidneys.** Venous pulse transit time (VPTT), venous impedance index in liver (HVI) and kidneys (RIVI), uncomplicated pregnancies (UP), gestational hypertension (GH), late-onset preeclampsia (LPE) and early-onset preeclampsia (EPE), at the level of the liver **(panel A)**, right kidney **(panel B)** and left kidney **(panel C)**.

**Figure 2:**
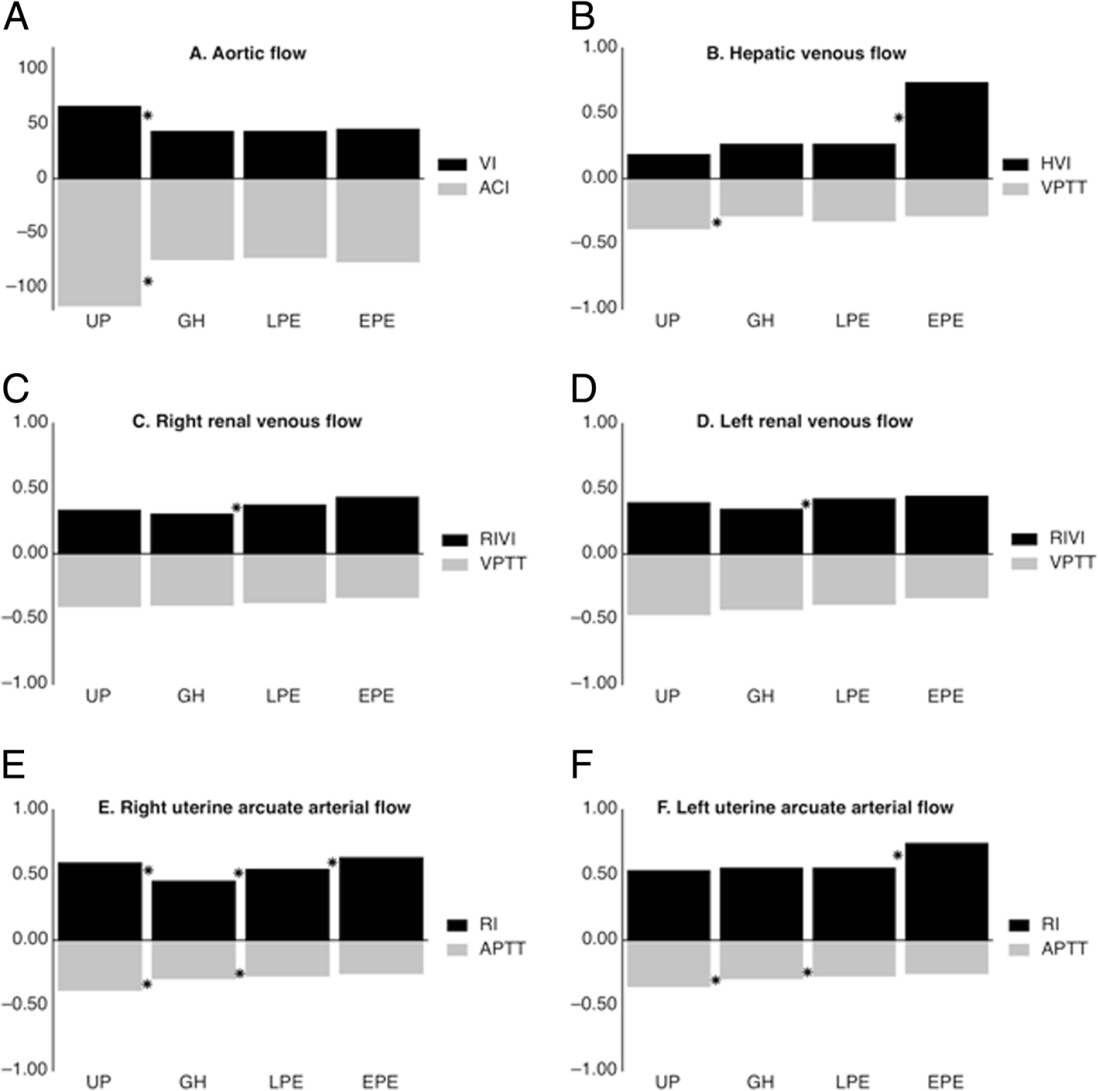
**Comparison of cardiac, arterial and venous hemodynamic parameters between uncomplicated and hypertensive pregnancies.** Parameters at the level of aorta **(panel A)**, liver **(panel B)**, right kidney **(panel C)**, left kidney **(panel D)**, right uterine arcuate artery **(panel E)** and left uterine arcuate artery **(panel F)**. Uncomplicated pregnancy (UP), gestational hypertension (GH), late-onset preeclampsia (LPE) and early-onset preeclampsia (EPE), VI = aortic flow velocity index, as measured by impedance cardiography (ICG), ACI = ICG-measured aortic flow acceleration index; HVI = hepatic vein impedance index, RIVI = renal interlobar vein impedance index; VPTT = venous pulse transit time, APTT = arterial pulse transit time. An asterisk indicates a statistically significant difference at nominal level α ≤ 0.05 with Mann–Whitney U test between UP and GH, between GH and LPE or between LPE and EPE.

There was a negative correlation between venous impedance index (HVI and RIVI) and VPTT for all groups at the level of liver and right kidney. For the left kidney, however, the negative correlation between RIVI and VPTT was only true for EPE, but not for UP, GH and LPE. The difference in slope and correlation between left kidney EPE and UP, GH or LPE was significant (*P* ≤ .05). Figure 1 shows the correlation between venous impedance index and VPTT for EPE, LPE and GH at the level of liver (Figure 1A), right (Figure 1B) and left kidneys (Figure 1C).Figure 2 illustrates the comparison between UP and GH, between GH and LPE, and between LPE and EPE for left ventricular systolic function (aortic flow), venous hemodynamic function at the level of hepatic, right and left renal interlobar veins and arterial hemodynamic function at right and left uterine arcuate arteries. Aortic flow parameters ACI and VI were lower in the hypertension groups than in UP, but were not different between GH, LPE and EPE (Figure 2A). The same was true for hepatic VPTT (Figure 2B). HVI was higher in EPE than in the other 3 groups (Figure 2B). RIVI were not different between UP and GH, or between the hypertension groups. However, RIVI was higher in the preeclampsia groups than in GH and UP (Figure 2C and D). Uterine arcuate APTT in GH was shorter than in UP and higher than in LPE, but not different between PE groups (Figure 2E and F). Uterine arcuate RI was higher in EPE than in other groups (Figure 2E and F).

## Discussion

From the results of this observational cohort study, some characteristics of cardiac, arterial and venous function can be deduced for gestational hypertension, late-onset preeclampsia and early-onset preeclampsia. In this so-called cardiovascular profiling, it is illustrated that: (1) gestational hypertensive diseases GH, LPE and EPE show impaired central arterial hemodynamic function as compared to UP, (2) arterial vascular tone in LPE is higher than in GH, but lower than in EPE, and (3) compared to GH, preeclampsia exhibits higher RIVI in association with proteinuria.

Our study is original because it evaluates the global maternal circulation, including heart, arteries and veins. The non-invasive methods used have all been thoroughly evaluated, and reproducibility and repeatability have been reported. Finally, the patients included in each group strictly comply with all reported criteria for each disorder, and are free of interfering maternal or gestational diseases.

Our study includes for each group a rather limited number of patients, specifically in the uncomplicated pregnancy group, which may imply a lack of power to show significant difference. Next to this, our study does not allow drawing definitive conclusions on the individual role of heart, arteries and veins in the development or the clinical presentation of PE, nor on the role of the venous compartment in PE-related organ dysfunction. To evaluate this, more clinical and experimental research is needed. Finally, the definition of preeclampsia as proteinuric gestational hypertension, as used in this paper, is not fully in line with the current definition of the American College of Obstetricians and Gynecologists’ Task Force on Hypertension in Pregnancy, stating that proteinuria is not a fundamental prerequisite of preeclampsia anymore.

### Venous hemodynamic dysfunction

Venous hemodynamic dysfunction in PE was reported for the first time by Bateman et al. [25]. In former publications, we have reported that RIVI is higher in EPE than in LPE [9] and that HVI is also increased in PE, as compared to UP [26]. Current data add to these reports that both EPE and LPE differ from GH in increased RIVI (Table 3). Increase of RIVI for both kidneys is associated with > 300 mg/24 h proteinuria and with increased serum uric acid concentrations (Table 1). RIVI is defined as (max velocity – min velocity)/max velocity. Minimum venous flow velocity has been linked to a sudden deceleration of forward flow due to the counteractive force of the right atrial systole, which causes intravenous backflow into the venous system by lack of valve between atrium and vena cava [27]. At the level of HV, this counteraction is responsible for reversal of the venous A-wave, leading to a triphasic pattern of HV Doppler waves in PE [26]. At the level of RIV, this counteraction is responsible for the so-called venous pre-acceleration nadir (VPAN) in PE, which is rarely seen in UP or in non-pregnant condition [9]. As such, these observations illustrate that intravenous backflow from atrial systole in PE is transported in retrograde direction up to the level of the renal parenchyma. Our current data illustrate that RIVI values in EPE and LPE are higher than in GH, implying that this intrarenal backflow seems to be an intrinsic feature for PE but not for GH (Figure 1, Table 3).

### Venous impedance and venous tone

An inverse correlation between venous impedance index and VPTT is illustrated in Figure 2. Pulse transit time is considered a measure for vascular tone: in conditions of increased vascular tone or stiffness, the propulsion wave is transported faster through the circulation than in conditions of low vascular tone, and this is responsible for a shorter time interval between ECG and pulse or Doppler wave [28-30]. During PE, shorter pulse transit times have been measured at both the arterial [31] and venous sides of the circulation [15]. We reported an increase of VPTT in UP and a reduction in PE, which correspond with a reduction or increase of venous tone in normal gestation and PE respectively [15]. Figure 2 illustrates the negative correlation between VPTT and venous impedance index, which accounts for UP, GH, LPE and EPE in liver and right kidney, but only for EPE in the left kidney. It is likely that increased venous tone in PE is associated with a faster and more distant rebound of atrial contraction throughout the venous circulation, up to the level of the kidneys. Figure 2 suggests that only in EPE, this rebound reaches the left kidney, which is more distant from the heart than the liver and right kidney [27]. As such, increased RIVI, with or without VPAN, can be considered a reflection of pulsatile counteraction of forward venous flow from the kidneys, which intermittently counteracts renal outflow during each atrial contraction [10]. Obstruction to renal venous efflux is known to induce proteinuria, both under experimental conditions [32] as in clinical syndromes such as renal vein thrombosis [33], the nutcracker syndrome [34] and the cardiorenal syndrome [35]. Whether this pulsatile rebound of atrial contraction is the trigger for renal dysfunction with increase of serum uric acid and occurrence of proteinuria > 300 mg/24h should be evaluated in further experimental and clinical research.

### Peripheral arterial hemodynamic dysfunction

Arterial hemodynamic dysfunction in severe preeclampsia usually presents clinically as overt arterial hypertension, related to vasoconstriction of smaller peripheral arteries and increased systemic vascular resistance. This can be associated with increased stiffness of the larger arteries [36]. Reduced arterial compliance persists for years in women who had pregnancies complicated with PE [37] and predisposes to gestational hypertensive complications when present before conception [38]. Arterial hypertonia and serum concentrations of vasoactive agents are reported to be higher in EPE than LPE, both during as before clinical onset of disease [3,4,39]. Our data correlate with this: as illustrated in Table 3, uterine arcuate arterial RI is higher in EPE than in LPE, which in turn shows shorter APTT than in GH.

### Central arterial hemodynamic function

Aortic flow parameters VI and ACI directly relate to cardiac systolic function and aortic compliance: the stronger the ventricular ejection and aortic compliance, the higher maximum velocity and acceleration of blood flow in the aorta. Reduced VI and ACI in gestational hypertensive diseases, as measured by ICG (Table 2), can be considered the results of impaired central arterial hemodynamic function. Impaired systolic function has been associated with atypical gestational morphologic remodelling of the heart [40] and is reported for GH [41], for preterm [42] and for term PE [7]. This dysfunction also persists for years after PE [43].

## Conclusions

The results of this study confirm those of other publications that cardiac and arterial function is different between gestational hypertensive diseases and normal pregnancies. Our study adds to this knowledge that venous hemodynamic dysfunction in PE is more pronounced than in GH. Because presence or absence of proteinuria is the fundamental difference between GH and PE, this venous dysfunction may perhaps be much more important in the clinical presentation of PE than currently considered today.

## Competing interests

The authors declare that they have no competing interests nor patents pending. They have received no financial funding from any company or institution.

## Authors’ contributions

WG participated in the design and coordination of the study and wrote the manuscript. KT, AS and JO carried out the Doppler ultrasonography and impedance cardiography and participated in the statistical analysis. TM participated in the study design and helped to draft the manuscript. GM participated in the statistical analysis. All authors read and approved the final manuscript.

## Pre-publication history

The pre-publication history for this paper can be accessed here:

http://www.biomedcentral.com/1471-2393/14/212/prepub
